# The Application of Image Processing Technology in Camera Picture

**DOI:** 10.1155/2022/9899610

**Published:** 2022-06-30

**Authors:** Yun Hong

**Affiliations:** Xi'an International University, Humanities and Arts College, Shaanxi, Xi'an 710077, China

## Abstract

The application scene of camera pictures in our life is very huge, and the effect of information conveyed by images is more intense than words and language, so it is very important for us to make good use of pictures obtained by photography or even pictures obtained by other means. Aiming at the processing problem of camera pictures, we have adopted image scaling method, camera picture color change method, gray degree processing method, image color space change method, image brightness processing method, and other methods to solve the processing problems of camera pictures. After reasonable processing, the presentation effect of camera pictures will be greatly improved, and the utilization efficiency will be greatly improved.

## 1. Introduction

In this paper, based on the processing technology of camera pictures, all kinds of camera pictures or other image resources we get in our lives need us to process certain data. If we make good use of ways and means, the value of original film samples can become higher, which provides great convenience and help for our related research and life. We mainly digitize the attributes of photographic images and then use related algorithms to change the target values to achieve our goals and find the changing rules of other indicators of the images with changed attributes. One of the goals of this paper is an overview of the latest technologies for PDE-based image enhancement and smoothing methods. A unified description of the basic idea is that the generalization and application of relevant values and results can find historical remarks and the policy of opening up problems. Another goal is to propose an in-depth processing method and an interesting parabolic-like equation that can bridge the gap between the problem and the recovery method [1]. In this paper, one-dimensional interpolation function and two-bit separable extended application image data are derived. Cubic convolution interpolation has ideal characteristics and is very useful in image processing. This technique can be implemented stably and effectively in digital computers and related problems in image processing [2]. The research based on morphology needs to design a computer-based image processing algorithm to reduce the background noise to enhance the image and propose a robust segmentation algorithm to extract geometric features, compactness, axial ratio, and so on. According to the results of image processing, classification and comparison can be carried out [3]. In this paper, a new low-level image processing technology is proposed, which mainly deals with edge and corner detection and keeps the noise reduction of the structure when processing the image. Nonlinear filtering is used to define the image part and pixel correlation. The new feature detector is based on the minimization of the local image area, and the measured area will be used as the smoothing field [4]. One of the aims of modern microscopes is to quantify two-dimensional, three-dimensional, and four-dimensional phenomena in biology, medicine, and medical materials. The design of the IMAGIC-5 software system explains the requirements of this data processing, and the image processing is special. The image will include a multivariate statistical analysis of collected data, and the resolution will be significantly improved by using a new angle reconstruction method to determine the three-dimensional structure of amorphous molecules [5]. Image processing must meet a series of formal requirements, architectural requirements such as locality, progressiveness, and causality of space, stability requirements, and morphological requirements, which correspond to shape-preserving properties (rotation invariance and scale invariance). A complete classification of all images with various scale changes meeting these requirements is given [6]. This text provides depth and extensive coverage of all topics encountered in the field of image processing and machine vision and can be used as a readable reference for all groups of people. It is not just image processing and machine vision courses but also makes difficult concepts easy to understand and provides a large number of carefully selected problems and examples for learning and reference [7]. In a display system for the display of a graphical object generated by a machine screen, the graphs to be displayed require data of a standardized representation in a memory, a position between the graphs displayed in the machine, a horizontal width, an angle of rotation, a brightness of the graphs, and an inverse weight step value between the graphs [8]. Interconnectivity between digital cameras and other devices is one of the major problems for consumers and digital camera manufacturers. Interconnectivity allows for more user-friendly use, appropriate application layer software, and digital photos to be sent to target disk storage, printers, websites, e-mail messages, or prints [9]. In a display system for generating a graphic object on a camera screen, there is a need for data [10] that can convert a specification and perform a specification in a memory, an initial position at the start of each conversion between an identified camera picture and the object, a conversion width, a brightness in the image object, and a weight step value corresponding to the conversion width. With appropriate application layer software, digital photos can be sent directly from the camera to the desired target. The interconnection function of the digital camera allows consumers to use the function to take pictures more conveniently, using specially designed communication protocols and picture transfer protocols [9]. The digital camera inputs the captured image data from the key input unit to the e-mail address data, stores it in the flash memory so that the data are associated with each other, and then transmits the image data and the address data to the computer through the port and the infrared communication unit [11]. In a camera system of an image sensor apparatus for sensing and storing images, a professional processing apparatus processes uploaded sensed pictures. The supply device print head senses the image and prints on a print medium stored inside the camera system [12]. An electronic device can send viewpoint information to a server, and a search of an image database in the server can find pictures taken from relevant viewpoints and can provide a user with an option to save or pair multiple images instead of images captured by the electronic device [13]. A panoramic moving image camera for continuously exposing a film in a photographic picture includes a fixed housing portion and a rotating housing portion and comprises a lens device, a main film sprocket, a feed sprocket, a bow reel coaxially arranged in a spaced planar area, and a motor device for providing the main film sprocket in a fixed housing portion [14].

## 2. Image Processing-Related Operations

### 2.1. Image Size Processing

It is not very difficult to get pictures, and the pictures we get are processed in the planning so that we can better achieve the desired effect. If the size is inappropriate, we should change the size of the image. Particularly, the photos taken by high-position digital cameras are often too large to be reasonably inserted into media courseware. If we do not insert it according to the requirements, it will make the courseware resources occupy more space. At this time, we need to compress the pictures and process them in many ways. We can use algorithms to modify the attributes of the pictures and process the color attributes, brightness, and grayscale in the right positions. If you need to crop the image, you can also use Photoshop tools to crop the image size. Of course, if the size of the processing can be used in a variety of ways, using software facilities and hardware facilities, plus appropriate algorithms to deal with the size of the picture, can achieve higher requirements.

### 2.2. Image Watermarking

In many scenes, when we use certain pictures as materials or need to show them in public, most of the images with watermarks obtained in the network will bring us some troubles. With the continuous development of computer networks and the continuous enrichment of knowledge and information resources, the difficulty of obtaining resources is declining, and watermarking processing is also an urgent need. We can still use Photoshop technology for processing, and the repair brush tool can color the redundant marks on the image, so that the marks are close to the main colors of the picture, so that the marks can be integrated into the image. But sometimes when we need to add our own watermark to the picture, we can also operate the picture, select the text tool in Photoshop to fill in the watermark text we want, and set the attributes such as transparency to add the watermark to the picture perfectly.

### 2.3. Photographic Image Processing

We need to solve the problem of white spots in the postprocessing camera pictures. The grayscale table has a range, and the brightest point is the endpoint of the range that can be debugged. The maximum hue value is 255. The maximum grayscale is set to pure white without other details. The color temperature of the light source needs to be considered when defining white. Whether in shooting or postprocessing, it is to restore the true color of the image. The visual elements of photographic pictures include composition, color, and form. Visual language uses corresponding patterns to draw people's emotions, and concrete or abstract elements are used as media to show the author's inner feelings.

(1) The purpose of photographic pictures of life comes from recording life. Photographers have different purposes for different feelings, which can be to restore the truest shooting moment or to make the photographs more aesthetic in a specific direction and have different tastes. (2) Photographic pictures used in commercial nature need more professional technical processing, from the aspects of tone, sharpness, saturation, how to cut, secondary composition, black-and-white processing, color temperature, exposure, and so on, so as to achieve the eye-catching effect with the best quality; thus, better publicity can be carried out.

## 3. Overview of Image Processing Algorithms

### 3.1. Image Color Space Transformation Method

In order to change the image, we can choose to change the basic hue of the image to achieve the effect of independent components of the color space of the picture. The effect of Lab color space is better than that of RGB, which cannot describe the visual effect of color quantitatively, so it is necessary to transform them into space. By analyzing the color of natural images, the statistical distribution results are transformed into uniform color space Lab in statistical sense. The orthogonal linear change is used to transform the red, green, and blue signals that can be felt by the human visual system into three irrelevant color variables. Matrix calculus is given here to realize the spatial transformation from RGB space to Lab, and the spatial transformation relationship of color is as follows:

(1) $$\begin{array}{c} \left[\begin{array}{c} x\\ y\\ z \end{array}\right]=\left[\begin{array}{ccc} \text{0.}412245 & \text{0.}357580 & \text{0.}180423\\ 0.212671 & \text{0.}715160 & \text{0.}072169\\ 0.019334 & \text{0.}119193 & \text{0.}950227 \end{array}\right]\left[\begin{array}{c} R\\ G\\ B \end{array}\right]. \end{array}$$

The values of each component in the XYZ space are changed to the corresponding Lab component values according to the function *f* (*x*) as follows:

(2) $$\begin{array}{c} \left\{\begin{array}{c} L{}^{*}=116f\left(\frac{Y}{Y_{n}}\frac{Y}{Yn}\right)-16,\\ a{}^{*}=500\left[f\left(\frac{X}{Xn}\right)-f\left(\frac{Y}{Yn}\right)\right],\\ b{}^{*}=200\left[f\left(\frac{Y}{Yn}\right)-f\left(\frac{Z}{Zn}\right)\right]. \end{array}\right) \end{array}$$

The above *F* (*x*) specific function is described as follows:

(3) $$\begin{array}{c} f\left(t\right)=\left\{\begin{array}{cc} t^{1/3} & \text{if}t>{\left(\frac{6}{29}\right)}^{3}\\ \frac{1}{3}{\left(\frac{29}{6}\right)}^{2}t+\frac{4}{29} & \text{otherwise} \end{array}\right\}. \end{array}$$

*L* ^*∗*^, *a*^*∗*^, and *b*^*∗*^ are the last color space values, XYZ is the conversion value of RGB after XYZ, and the conversion from Lab to RGB color space also needs XYZ as the basis and then changes the color space. The component of Lab needs to change according to the function and then become the component of XYZ as follows:

(4) $$\begin{array}{c} \left\{\begin{array}{c} X=X_{n}f^{-1}\left(\frac{1}{116}\left(L{}^{*}+16\right)+\frac{1}{500}a{}^{*}\right),\\ Y=Y_{n}f^{-1}\left(\frac{1}{116}\left(L{}^{*}+16\right)\right),\\ Z=Z_{n}f^{-1}\left(\frac{1}{116}\left(\left(L{}^{*}+16\right)-\frac{1}{200}b{}^{*}\right)\right). \end{array}\right) \end{array}$$

After the above formula, the *f*^−1^(*x*) function is as follows:

(5) $$\begin{array}{c} f^{-1}\left(t\right)=\left\{\begin{array}{cc} t^{3}, & \text{if}t>\frac{6}{29}\\ 3{\left(\frac{6}{29}\right)}^{2}\left(t-\frac{4}{29}\right),\; & \text{otherwise} \end{array}\right). \end{array}$$

Each component of XYZ is converted to an RGB component as shown in the following formula:

(6) $$\begin{array}{c} \left[\begin{array}{c} R\\ G\\ B \end{array}\right]=\left[\begin{array}{ccc} 3.240479 & -1.537150 & -0.498353\\ -0.969256 & 1.875992 & 0.041556\\ 0.055648 & -0.204023 & 1.057311 \end{array}\right]\left[\begin{array}{c} X\\ Y\\ Z \end{array}\right]. \end{array}$$

### 3.2. Image Relational Mapping Processing

After having the color table of the template and the color table of the target picture, we need to consider which colors and how to use the matching relationship to allocate the color table. For the purpose of reconstructing the image color selected in the template image guide, the matching color table must also consist of the color of the target image as much as possible. The relationship of color composition is divided into visual contrast between colors, proportion of color distribution, coordination of color matching, and so on. According to the relationship between the three-dimensional color of the object scene image, we can define the part which is suitable for the large range of color transformation according to the color correlation between the object scene image and the background image and adjust the attribute between the object color correlation image and the object color table from different angles. Visual difference relationship between *M*={*C*_1_, *C*_2_,…, *C*_*n*_} colors in the target color table, relationship between color tables *M* and *C*, and relationship between different color points are described as follows:

(7) $$\begin{array}{c} \left\{\text{mean}\left(M\right)⟶c_{i};i,j\in n\&\&i\neq j.C_{i}⟶C_{j}\right). \end{array}$$

According to the purpose of color transition, by matching the visual difference relationship between the color table and the target color table, the visual difference relationship between the target colors is maintained. In the separation of foreground and background, the ratio of the total number of pixels of a certain color to the total number of pixels of the foreground is adopted to maximize the proportion of the color of the matching template image in the target image. A matching color table is generated according to the brightness rules in the target color table. When creating a new set of color tables, you need to adjust the proportion of the color tables matched with the selected color table according to the brightness distribution of the color table and adjust and calculate the proportion between the matched color table and the selected color table according to the brightness distribution of the color table. The figure is defined as follows for *E*_*p*_:

(8) $$\begin{array}{c} E_{p}=\sum_{i}^{n}\left\{T_{i}|M_{i}\right\}. \end{array}$$

*T*_*i*_, *M*_*i*_ represents a certain element, where *n* represents the number of elements. Visual difference is the main factor affecting the generation of matching color table. If you need to maintain the visual differences of the color table, you need to maximize the similarity of the contrast matrix. Make sure that the visual difference between the two color tables is much larger than the previous data. The comparison matrices of the two color tables need to be standardized after interpolation and comparison:

(9) $$\begin{array}{c} C{}^{*}=\frac{c-u}{\delta }. \end{array}$$

*C* ^*∗*^ is our standard value after processing, *C* is the value before processing, *u* is the mean of all samples, *δ* is the standard deviation in all data, and the root mean square error of normalized values of all data is used to measure the visual similarity between two color tables:

(10) $$\begin{array}{c} E_{c}=\sqrt{\frac{\sum_{i=1}^{n}\left|C{}^{*}\right|}{n}}. \end{array}$$

If we consider the contrast of the color table from another aspect, we cannot grasp the overall effect of the color table as a whole. We need to pay attention to the visual difference between the color points in the table and the overall average value. The total contrast of the color table is

(11) $$\begin{array}{c} E_{ca}=E_{c1}+E_{c2}. \end{array}$$

In this formula, *E*_*c*1_ is the contrast difference representing the average value *M* in the color table, *E*_*c*2_ is the contrast difference between the color points in the color table *M*, and quantizing the color table matching effect into the energy value *E* can be recorded as

(12) $$\begin{array}{c} E=E_{p}+E_{ca}. \end{array}$$

### 3.3. Adaptive Processing of Picture Brightness

The background color attribute of the scene image is simplified, and the overall brightness is changed to adapt to the desired effect. In particular, a color template provided to a landscape image within a camera screen cannot easily find a color suitable for the background image of the scene. The color processing of the background part is divided into two types: one is to specify the corresponding color interactively, and the other is to adjust the color adaptively. Our brightness processing must keep the relationship between the parts of the object image and the brightness adjustment function:

(13) Itl−ctlptl−Itl=Iml−CmlPml−Iml.

After scene image segmentation, the subimages are processed with heavy color. Image segmentation has certain particularity, which cannot achieve complete and accurate segmentation. In the combination process of each image, color jump is easy to occur. Partial overlap and partial information loss will occur in the part near the edge. In addition, there will be blank points of information loss in the edge points of the reconstructed map, and the migration of combined colors between segmented submaps is not smooth. To solve the manhole problem, the first problem to be solved is to distinguish the manhole types. The edge position of the reconstructed image is misaligned and missing to a certain extent, and the edge position of the subimage is determined by the edge pixels of the target image. The edge detection algorithm is used to realize the edge detection of the object image. The probability of judging a pixel as a boundary point is described as the edge intensity of the pixel, that is, the brightness value of the edge detection image. Longitudinal arrangement and transverse arrangement are cavity point arrangement. It can be determined that a cavity point may be an edge point by calculating an edge intensity between a plurality of pixels in the arrangement gradient direction of the cavity point as follows:

(14) $$\begin{array}{c} \text{Longitudinal}\left\{\begin{array}{c} \text{max}\left(\phi \left(w1\right),\phi \left(h2\right),\phi \left(w2\right)\right)==\phi \left(h2\right),h2\text{Isanedgepoint,}\\ \max\left(\phi \left(l1\right),\phi \left(h2\right),\phi \left(w2\right)\right)\neq \phi \left(h2\right),h2\text{Non}-\text{edgepoint.} \end{array}\right) \end{array}$$

When we may face the problem of color jump, we need to consider the problem of partial overlap and information loss near the edge SSO, which is the edge point, retrieve the blank points lost in the edge points of the reconstruction image, and determine the edge position of the subimage and the edge pixel of the target image SSO, so that the migration between the combined colors of the segmented subimages can be smoother.

(15) $$\begin{array}{c} \text{Lateral}\left\{\begin{array}{c} \max\left(\phi \left(l1\right),\phi \left(h2\right),\phi \left(l2\right)\right)==\phi \left(h2\right),h2\text{Isanedgepoint,}\\ \max\left(\phi \left(l1\right),\phi \left(h2\right),\phi \left(l2\right)\right)\neq \phi \left(h2\right),h2\text{Non}-\text{edgepoint.} \end{array}\right) \end{array}$$

### 3.4. Image Processing Experiment

Color conversion has important application value. The color region is selected according to the interaction between the input target image and the template image, and the color of the input target image is added by using the color average value. Image color transfer effects corresponding to different regions of the target image are interactively selected for the input template image, details of which are as follows:

(16) $$\begin{array}{c} l=\left\{\begin{array}{c} T1⟶R1\\ T2⟶R2\\ T3⟶R3 \end{array}\right\},\left(T1,T2,T3\right)\in T. \end{array}$$

According to formula (16), we can find the selected color area for interaction between the target image *T* and the template image *R* and add the color of the input target image *R* to the image *T* by using the color average value. If formula (16) is missing, the interaction mode is unknown, and the transmission effect of the corresponding image cannot be observed.

### 3.5. Power Consumption for Pixel Processing

In order to optimize the actual power consumption of the shooting equipment on the screen, it is necessary to establish a power consumption evaluation model of the shooting screen and then evaluate the power consumption of the shooting image in the later stage. The OLED screen displays colors through three independent light sources, and the function of calculating the power consumption per pixel of the screen can be described as follows:

(17) $$\begin{array}{c} E_{\text{scrrrn}}\left(r,g,b\right)=\left(f\left(R_{i}\right)+f\left(G_{i}\right)+f\left(B_{i}\right)\right). \end{array}$$

The left side of the equation is the power consumption of each pixel of the picture when emitting light, and the right side is the power consumption of red, green, and blue color components and the power consumption of the whole image processing:

(18) $$\begin{array}{c} E_{\text{scrrrn}}=C+\sum_{i=1}^{n}\left(f\left(R_{i}\right)+f\left(G_{i}\right)+f\left(B_{i}\right)\right). \end{array}$$

Here, *C* is a constant; that is, according to the obtained data, the power consumption when displaying a black image on the screen is calculated, and a fitting curve is generated. The universal function fitting degree of color component of function fitting curve is as follows: red = 0.996, green = 0.969, and blue = 0.985. The starting point of image capture is the power consumption required when displaying black images on the screen, which is collectively referred to as the power consumption of image processing devices. The power consumption required by image processing equipment is constant, and the test value at this time is *C* = 0.5856 W. The overall trend of function adaptation image is that the power consumption curve increases with the increase of CL, basically at the same starting point, reflecting the power consumption value of camera equipment. The performance relationship is as follows:

(19) $$\begin{array}{c} \left\{\begin{array}{c} E_{\text{blue}}\gg E_{\text{green}}∼E_{\text{red}},CL\leq 17,\\ E_{\text{blue}}\gg E_{\text{green}}>E_{\text{red}},CL>17. \end{array}\right) \end{array}$$

The power consumption of blue is the largest among the three types. The power consumption curves of the front green and red treatments are relatively close, and the back part of green is obviously greater than red.

### 3.6. Color Reconstruction of Image

Through the corresponding reconstruction algorithm, the input image *I* is repainted and then output, so that the processed image meets a better image visual effect. The color channel separates *I*={*I*_*r*_, *I*_*g*_, *I*_*b*_} and converts *I*={*I*_*r*_, *I*_*g*_, *I*_*b*_} into the HSL color space. According to each subgraph of the color table, each subgraph is converted into a component gallery *C*, and the RGB component corresponds to each gallery:

(20) $$\begin{array}{c} \left\{\begin{array}{c} Ir\text{C}\\ Ig⟶\\ Ib \end{array}\right)\left\{\begin{array}{c} Irc1,Irc2,\ldots Ircn,\\ Igc1,Igc2,\ldots Igcn,\\ Ibc1,Ibc2,\ldots Ibcn. \end{array}\right) \end{array}$$

The goal of color reconstruction is to select the corresponding RGB component mapping from *C* and to ensure that *I*, *J*, and *K* are not equal while minimizing the power consumption of the combined image.

### 3.7. Image Color Table Generation Method

The color table *C* is made by image clustering. This method is a common method because of the large amount of computation, the results obtained by the clustering method are simple and not inclusive, and the visual distance between colors cannot be guaranteed. Therefore, it is of great significance to find a variety of color table synthesis methods that can not only reduce the amount of calculation but also ensure the visual distance between colors. CIELab is abstracted as a three-dimensional sphere, and the radius of the sphere is set to *r* = 100 (corresponding to the color space). The possible ranges of *a* and *b* vary depending on the value of *L*, and the origin of the coordinate system coincides with the center of the sphere. Since the range of *Z* coordinates (−50, 50) and *L* (0, 100) of the sphere is different, the input value of *L* must be converted and input *L* to calculate the *xL* value:

(21) $$\begin{array}{c} \left\{\begin{array}{c} \frac{xl}{100}=\frac{l-50}{50},L>50,\\ \frac{100-xr}{100}=\frac{L}{50},L\leq 50. \end{array}\right) \end{array}$$

It takes a lot of computation to get the set of color combinations by image clustering. The clustering method is not inclusive, and the visual distance between colors is not guaranteed. The significance of formula (21) lies in reducing the calculation amount and ensuring the visual distance between colors. CIELab is abstracted into a sphere, and the range of related attributes is set. After that, it is very convenient to operate according to the formula.

When the value of *L* is determined, the cross-sectional range of the corresponding color space is determined, and the radius of the cross-sectional circle is calculated:

(22) $$\begin{array}{c} r={\sqrt{R^{2}-\left(xr\right)}}^{2}. \end{array}$$

Map the points on the coordinate system to CIELAB by selecting the difference between the points on the coordinate system and the CIELAB value range, as follows:

(23) $$\begin{array}{c} \left\{\begin{array}{c} \left\{\begin{array}{c} \frac{x}{100}=\frac{a}{127},x\leq 0;\\ \frac{x}{100}=\frac{a}{128},x>0; \end{array}\right)\\ \left\{\begin{array}{c} \frac{y}{100}=\frac{b}{127},y\leq 0;\\ \frac{y}{100}=\frac{b}{128},y>0; \end{array}\right) \end{array}\right)\left(x⟶a\right)\left(y⟶b\right). \end{array}$$

It is judged that the newly generated color point *P* satisfies the relationship of the color point set *D*:

(24) $$\begin{array}{c} \left\{\begin{array}{c} d\left(i\right)\left(L_{i,}a_{i},b_{i}\right)\in D;\\ Ed_{i}=\sqrt{{\left(L-L_{i}\right)}^{2}+{\left(a-a_{i}\right)}^{2}}+{\left(b-b_{i}\right)}^{2},\\ Ed_{i}\geq E0. \end{array}\right) \end{array}$$

### 3.8. Color Table Combination Algorithm

The combination algorithm solves the problem of selecting the best combination of subimages from component library *R* and converts each component of the target image according to the color table hue to generate the corresponding color component library. Each color channel has a sublibrary of corresponding components, and the number of images in the sublibrary is the number of colors in the reference color table. The power consumption of each subgraph is calculated by using the power consumption evaluation model, and the power consumption matrix of each subgraph is established:

(25) $$\begin{array}{c} E_{\text{matrix}}=\left[\begin{array}{cccc} E_{r1} & E_{r2} & \ldots & E_{rn}\\ E_{g1} & E_{g2} & \ldots & E_{gn}\\ E_{g1} & E_{g2} & \ldots & E_{gn} \end{array}\right]. \end{array}$$

*E*_matrix_ elements in the same column have the same hue, while elements in the same row correspond to the color table one by one, and the RGB component area of the target image is represented by different lines. Select three factors from *E*_matrix_ to keep the combined power consumption optimal. The selected element must satisfy the restriction, and the relationship is as follows:

(26) $$\begin{array}{c} \left\{\begin{array}{c} E_{ri},E_{gi},E_{bk},\\ \text{min}\left(E_{ri}+E_{gi}+E_{bk}\right),\\ i\neq i\neq k. \end{array}\right) \end{array}$$

## 4. Processing Methods of Photographic Pictures

### 4.1. Image Scaling Method

**Figure 1:**
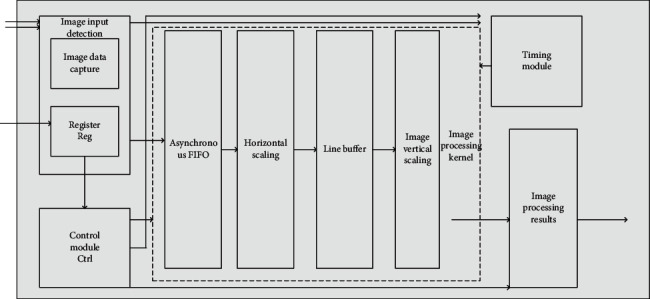
Image data and hue operation flow.

We have a certain operation and arrangement for picture processing. We can separate the processing of pictures independently and implement single operation after dividing them into several types of modules. Image data scaling and tone modification need the help of algorithms; in the case of interpolation points will not change, we need to change the interpolation according to different and relatively appropriate algorithms, using filters to achieve different image data processing under different algorithms. Compared with multiple data changes, the asynchronous FIFO of data buffer and LineBuffer of line buffer are of appropriate size, which can realize data migration and change of scaling ratio. The timing control module is used instead of the traditional MCU module, which saves some resources. In the system, the image data is input and then detected (including high-precision capture of the image data, the configuration parameters are stored in the storage area), and the control block Ctrl, the multilevel scaling kernel (asynchronous FIFO, horizontal and vertical scaling, and line memory), the timing module, and the processing contents after data modification are shown in Figure 1.

According to different and relatively appropriate algorithms, the interpolation is changed, and the filter is used to realize the processing of picture data. Because the data needs to be changed many times, the data buffer asynchronous FIFO and the line buffer LineBuffer can realize the data migration and the change of scaling ratio, and the timing control module can replace the traditional MCU module, which can improve the efficiency.

**Figure 2:**
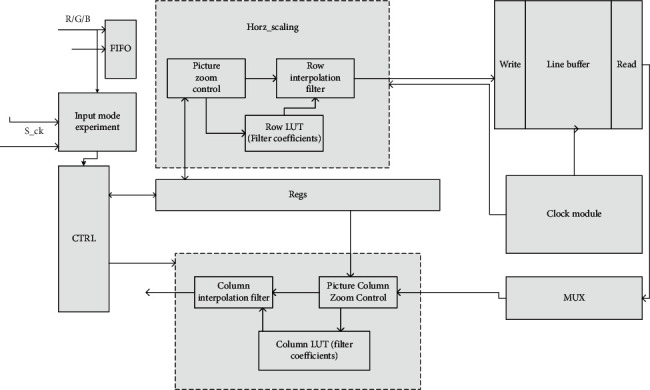
Scaling core processing flow.

Image scaling kernel processing: according to the above horizontal structure of the secondary image processing scaling kernel architecture, you can give the specific operation division of the core scaling module, as shown in Figure 2.

We input the detected image signal to identify its color coding format and the polarity of horizontal and vertical synchronization signals, send the calculated information to the receiving module, and control the scaling image processing according to different input information. At the same time, in order to save hardware resources while processing data in real time, the calculated data should be stored in ROM in advance, and the filter coefficients corresponding to configuration information should be found out in the table for interpolation processing. We input the detected image signal to identify its color coding format and the polarity of horizontal and vertical synchronization signals. The filter coefficients of the corresponding configuration information are found out in the table and provided for interpolation processing. The output images with different scaling ratios will be divided into many pixel blocks, which leads to the prominent mosaic phenomenon. The image quality is not very good; especially, the smaller the PSNR, the worse the imaging quality.

The output image with different scaling ratio will be divided into small pixel blocks, the mosaic phenomenon is prominent, the picture quality is not very good, and there will be pixel blocks when the scaling ratio is 0.5. Bicubic different scaling ratio display effect is good and according to the four-point two-segment interpolation effect will be better, using built-in functions to get objective scores of several algorithms, and in the case of magnification scaling ratio greater than 1, the larger the value of scaling ratio, the smaller the PSNR, the worse the imaging quality, and the smaller the scaling ratio less than 1 under reduced imaging. Scale ratio can be infinitely large in theory; most of the algorithms in the case of large scale ratio of the image imaging display effect is average.

The display effect of bicubic in different scaling ratios is OK. The nearest algorithm produces a block of pixels at a scale of 0.5, and therefore, compared with bicubic, the effect will be a little worse. The larger the scaling ratio obtained by using the four algorithms with built-in functions, the smaller the PSNR and the worse the imaging quality. In theory, it can be infinitely enlarged. In fact, the imaging display effect of superlarge scaling ratio images is also general.

For example, Tables 1 and2 are used to count the average scaling time required for image processing of different algorithms used in our experiments. According to the above data, we can see that the processing speed of bicubic interpolation is slower than that of the nearest algorithm, and it is the slowest. The processing speed of the bilinear algorithm is close to that of piecewise quadratic processing, and the speed of both is far higher than that of bicubic interpolation. According to the above discussion, the scaling effect of the piecewise quadratic interpolation algorithm is worse than that of the bicubic interpolation algorithm, and its advantages are obvious. It has good low-pass characteristics and will not be easily distorted. All the above algorithms need to be implemented according to hardware, and the scaling speed of picture processing is better than the typical picture processing scaling algorithm, so we need to compare and select the core picture processing scaling algorithm suitable for the scene ( Table 3).

**Figure 3:**
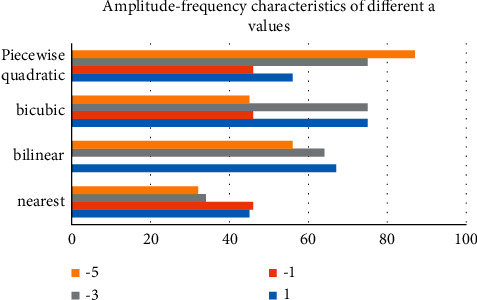
Amplitude-frequency characteristics of different parameter a under four algorithms in image processing.

Under various image processing scaling algorithm schemes, all four algorithms have their own degrees of freedom a. When calculating filter coefficients, only a and interpolation decimal offset are considered, and parameter a is adjusted to change the spectrum characteristics of the filter to suppress aliasing. After the algorithms are distinguished, the scaling processing effect of pictures will be different, and the processing speed and resource consumption will be different, as shown in Figure 3.

**Figure 4:**
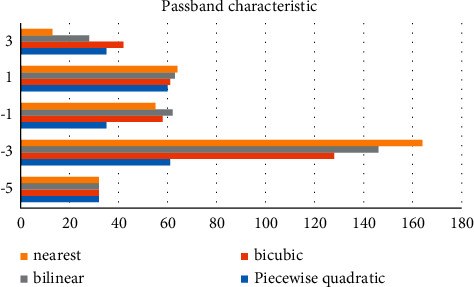
Passband characteristics under different parameter a.

After selecting different parameter a, the rendering effect of the image will be different. We will pay attention to the consumption of hardware while operating, and we need to concentrate on the performance of hardware facilities to process the image centrally. In order to process the image better and get a better visual effect, we need to know the passband characteristics. The comparison of passband characteristics is shown in Figure 4.

### 4.2. Color Change Method of Photographic Image

**Figure 5:**
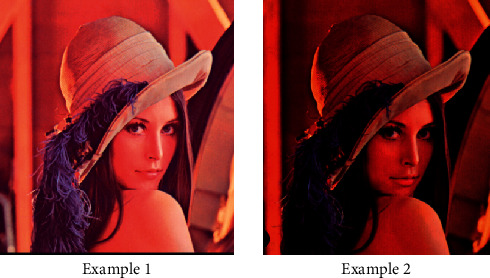
Color reconstruction picture.

Image color reconstruction algorithm is mainly used in image color conversion, and color reconstruction can make us change pictures more freely. Image reconstruction is quite different from ordinary attribute single change. Direct reconstruction is to change our key parts, and the degree is controlled by ourselves. A single image resource is not conducive to the efficiency evaluation of image color processing, and the algorithm will have shortcomings. We need to select multiple scene images and other template images to process the color and evaluate the efficiency of the algorithm. The input image is subjected to a color migration algorithm for about 10 times for an available template image, and finally, a large amount of data is analyzed. The color reconstruction operation of the camera image is in Figure 5.

**Figure 6:**
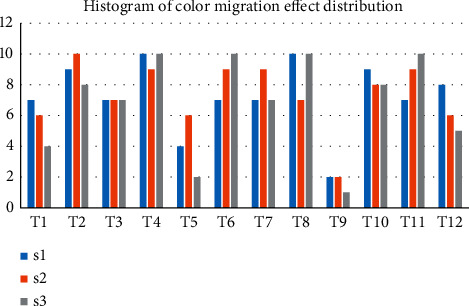
Color migration effect.

In the experiment, SSO is the image data we use as the template, SSO is our target image data, and using the algorithm can achieve the ideal migration probability of about 71%. Template image color transfer processing effect is not good, the same template image for different target image data has some differences, specifically, and the compatibility of image color has a great relationship, as shown in Figure 6.

**Figure 7:**
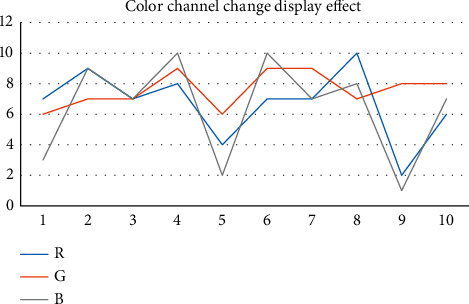
Color channel changes display effect.

The image is composed of pixels, and the color is expressed according to the actual situation of RGB components. Images of various RGB components can be used as the basis for testing. Because (0,255) is the value range of the color channel, it is actually difficult to compare the color difference by adding 1 color change. We cross eight color levels at a time, and the visual effect (roughly divided into five effect degrees, 1–5) changes roughly in the process of change. The RGB color component has 32 color levels (0–255 difference is 8 to 1). The RGB values of different levels are displayed on the hardware screen, so we need to switch and record the change of the display effect of image color transformation regularly. Finally, we need to repeat the operation to take the average value (about 10 times), as shown in Figure 7.

In the three numerical changes of *R*, *G*, and *B*, the regular increase of 8 orders of magnitude can make the change effect more gentle and the visual effect more intuitive (Figure 7). Comparing the change display effect is more convenient for us to summarize the rules and methods.

### 4.3. Gray Degree Processing of Photographic Images

**Figure 8:**
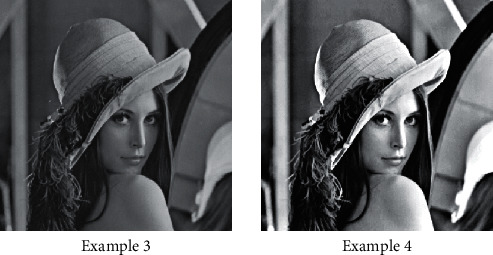
Grayscale processing picture.

Image processing algorithm plays a very important role in image processing. We can use it to process photographic pictures, which also has a great influence on the posteffect and quality effect of the processed photographic pictures. Our preprocessing algorithm mainly calculates and processes the grayscale of the images, which can improve the image contrast and other parameters to a certain extent. By changing the brightness and shade of different brightness areas and increasing the numerical distribution of image gray space, the contrast of the image in different situations can be greatly improved, and the foreground target can be greatly highlighted. The gray processing of the picture is shown in Figure 8.

**Figure 9:**
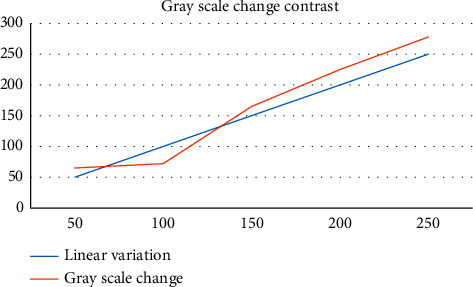
Comparison of image gray level change effect.

Linear transformation algorithm is one of the most basic algorithms in image transformation. The gray value of the initial image has a linear relationship with the gray value of the transformed image, as shown in Figure 9.

We need to consider that, in order to achieve the purpose of reconstructing the selected colors of the photographic picture template, the matched color table must be as similar to or even composed of the colors of the target image *M* as possible. The distribution of color table *C* also needs to grasp the relationship of color composition from multiple dimensions and also needs to adjust the attribute of *M* between the target color associated image C and the target color table from different angles so as to achieve the reasonable distribution of color table.

**Figure 10:**
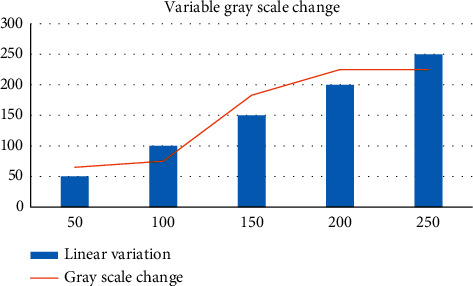
Image variability gray level change.

The gray space can change between 0 and 255. In order to prevent the grayscale from being displayed due to leapfrogging, we need to increase the contrast of different grayscale spaces in a certain application scene, compare with a single linear algorithm, and propose a method of different transformation functions in different grayscale intervals, so as to achieve the linear exchange of different grayscale intervals shown in Figure 10.

The gray level change of image has important significance and value. According to Figures 9 and 10, we can know that there are linear processing methods and nonlinear processing methods. The single linear algorithm is compared, and the linear exchange between different gray levels is realized. In the nonlinear change algorithm, the nonlinear function of power law change and exponential change is used to deal with the pixels of the image, which better avoids the distortion of the image. The single linear change is simple and convenient, and the nonlinear flexible change space is large. Both of them have their own advantages and have very important use significance.

**Figure 11:**
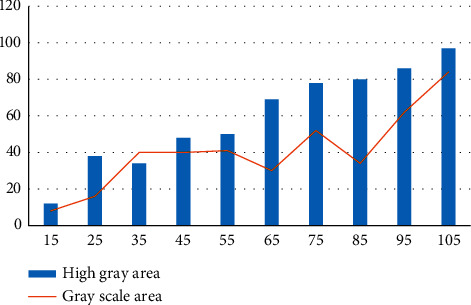
Image power change.

In the algorithm of image nonlinear change, the basic principle of nonlinear change is to use the nonlinear function of power law change or exponential change to process every pixel of the image. The logarithmic change firstly takes the original image as input and calculates the gray value of each pixel by logarithmic calculation. Due to the distribution characteristics of the exponential function, the exponential fluctuation amplitude in low-gray area is obvious, which can effectively improve the detail contrast in dark area of image. The operation of power law transformation takes the gray value of the image as the bottom of the power law function and other parameters as the coefficients and power values of power operation, which are similar to exponential changes to prevent image distortion. The change needs to be operated by power law transformation according to different parameters. When the parameter is less than 0, the power law transform can improve the contrast ability of dark area and ensure the color range of high-brightness area. When the input parameter is greater than 0, the switch can be changed to improve the intensity of the brightness area and the contrast of the high-brightness area so as to ensure that the image in the low brightness area is not distorted, as shown in Figure 11.

## 5. Concluding Remarks

The image processing technology written in this paper is applied to photographic images. We provide many aspects of image processing and how to make the image resources obtained by shooting use algorithms to change their attributes. For image processing is the focus of color and size processing, we can simply change not only the value of a single attribute but also the color reconstruction of large and free modification. Visually meticulous processing sometimes depends on contrast, and the difference is subtle, but the attribute of contrast is also very important. The contrast between the high-brightness area and the low-gray area will also have an impact on the final quality of the camera pictures in our hands, and the scaling of the image at the pixel block level should also be paid attention to. We know the rules clearly and master the methods so that we can better deal with the image processing problems.

## Figures and Tables

**Table 1:** PSNR index of each algorithm of bikes in standard diagram.

	P SNR-bikes/unit: dB				
Scale ratio	2	4	8	0.3	0.5
Nearest	31.2873	27.9418	23.9202	22.3221	20.7878
Bilinear	35.7905	30.8422	30.8422	25.0325	23.2467
Bicubic	37.6688	31.7796	31.7886	26.1759	23.5067
Piecewise quadratic	36.4299	31.2586	31.2696	25.8461	23.4166

**Table 2:** PSNR index of each algorithm of caps in standard diagram.

	P SNR-bikes/unit: dB				
Scale ratio	2	4	8	0.3	0.5
Nearest	25.8943	22.6036	20.2453	20.0272	19.0793
Bilinear	28.6147	24.9835	23.0147	22.9415	22.0353
Bicubic	31.3147	26.6023	24.0458	24.0612	23.8647
Piecewise quadratic	29.9941	25.5041	23.9498	23.8701	23.6724

**Table 3:** Statistics of average image processing time of each algorithm.

	Nearest	Bilinear	Bicubic	Piecewise quadratic

Processing reduction time/s	1.31	5.71	14.32	6.14
Processing amplification time/s	1.11	1.18	1.29	1.09

## Data Availability

The experimental data used to support the findings of this study are available from the corresponding author upon request.
